# ASSESSMENT OF ELECTROLYTE ABNORMALITIES IN OLDER ADULTS WITH COVID-19 DELIRIUM

**DOI:** 10.1093/geroni/igad104.3186

**Published:** 2023-12-21

**Authors:** Milenko Petrovic, Esther Park, Amanda Pangle, Jeanne Wei, Gohar Azhar

**Affiliations:** University of Arkansas for Medical Sciences, Little Rock, Arkansas, United States; University of Arkansas for Medical Sciences, Little Rock, Arkansas, United States; University of Arkansas for Medical Sciences, Little Rock, Arkansas, United States; University of Arkansas for Medical Sciences, Little Rock, Arkansas, United States; University of Arkansas for Medical Sciences, Little Rock, Arkansas, United States

## Abstract

This retrospective study explored the impact of COVID-19 on altered sodium and potassium in older adults with delirium. EMRs from the University of Arkansas for Medical Sciences (UAMS) database were reviewed from January 7, 2018 to January 5, 2021 for electrolyte abnormalities and delirium. Inclusion criteria were age 65 or older, all races, genders, and ethnicities with COVID or non-COVID infections. We identified 319 patients with COVID-19 out of which 108 (33.8%) had delirium during COVID. In COVID-delirious patients, hypokalemia was present in 8, hyperkalemia in 10, hyponatremia in 9, and hypernatremia in 20.127/319 (39.8%) COVID patients had an electrolyte disorder at some point with hypokalemia being most represented at 58/127 (45.7%). Out of the patients who experienced hypokalemia at some point, 34/319 (10.6%) had delirium. In another cohort of 306 older adults with non-COVID pneumonias we identified 173 patients with electrolyte disorders and 106 (34.6%) with delirium during pneumonia. Electrolyte disturbances were more present in non-COVID pneumonias vs. those with COVID (p< 0.001). Hypokalemia was more identified in non-COVID, 56/306 (18.3%), vs. COVID patients, 34/319 (10.6%) (p< 0.006). Interestingly, hypernatremia was greater in COVID groups (p< 0.0321). There was no difference in delirium between COVID vs non-COVID patients, but electrolytes disorders and hypokalemia were observed with greater frequency in non-COVID pneumonias. Notably, hypernatremia developed more in patients suffering from COVID and since it can be associated with seizures and cerebral hemorrhage, particular attention should be paid to prevent hypernatremia from developing in order to reduce COVID-related morbidity and mortality.

